# Mirizzi Syndrome—The Past, Present, and Future

**DOI:** 10.3390/medicina60010012

**Published:** 2023-12-21

**Authors:** Jonathan G. A. Koo, Hui Yu Tham, En Qi Toh, Christopher Chia, Amy Thien, Vishal G. Shelat

**Affiliations:** 1 Department of General Surgery, Tan Tock Seng Hospital, Singapore 308433, Singapore; jonathan.koo@mohh.com.sg (J.G.A.K.); huiyu.tham@mohh.com.sg (H.Y.T.); 2 Lee Kong Chian School of Medicine, Nanyang Technological University, Singapore 308232, Singapore; tohe0006@e.ntu.edu.sg; 3 Department of Gastroenterology and Hepatology, Tan Tock Seng Hospital, Singapore 308433, Singapore; christopher_tw_chia@wh.com.sg; 4 Department of General Surgery, Raja Isteri Pengiran Anak Saleha Hospital, Bandar Seri Begawan BA 1710, Brunei; amy.thien@moh.gov.bn

**Keywords:** Mirizzi syndrome, cholelithiasis, choledocholithiasis, cholecystectomy, subtotal cholecystectomy, laparoscopic, bile duct injury

## Abstract

Mirizzi syndrome is a complication of gallstone disease caused by an impacted gallstone in the infundibulum of the gallbladder or within the cystic duct, causing chronic inflammation and extrinsic compression of the common hepatic duct or common bile duct. Eventually, mucosal ulceration occurs and progresses to cholecystobiliary fistulation. Numerous systems exist to classify Mirizzi syndrome, with the Csendes classification widely adopted. It describes five types of Mirizzi syndrome according to the presence of a cholecystobiliary fistula and its corresponding severity, and whether a cholecystoenteric fistula is present. The clinical presentation of Mirizzi syndrome is non-specific, and patients typically have a longstanding history of gallstones. It commonly presents with obstructive jaundice, and can mimic gallbladder, biliary, or pancreatic malignancy. Achieving a preoperative diagnosis guides surgical planning and improves treatment outcomes. However, a significant proportion of cases of Mirizzi syndrome are diagnosed intraoperatively, and the presence of dense adhesions and distorted anatomy at Calot’s triangle increases the risk of bile duct injury. Cholecystectomy remains the mainstay of treatment for Mirizzi syndrome, and laparoscopic cholecystectomy is increasingly becoming a viable option, especially for less severe stages of cholecystobiliary fistula. Subtotal cholecystectomy is feasible if total cholecystectomy cannot be performed safely. Additional procedures may be required, such as common bile duct exploration, choledochoplasty, and bilioenteric anastomosis. *Conclusions*: There is currently no consensus for the management of Mirizzi syndrome, as the management options depend on the extent of surgical pathology and availability of surgical expertise. Multidisciplinary collaboration is important to achieve diagnostic accuracy and guide treatment planning to ensure good clinical outcomes.

## 1. Introduction

Mirizzi syndrome is a rare complication of gallstone disease, with a variable incidence of 0.3% to 1.4% in Europe and the United States of America, 4.7% in Mexico, and 5.7% in Chile [1]. It is caused by the obstruction of the common bile duct (CBD) or common hepatic duct (CHD) by external compression from an impacted gallstone in the neck or infundibulum (Hartmann’s pouch) of the gallbladder or within the cystic duct. The importance of this syndrome is related to its clinical presentation with jaundice and technical difficulty in identifying the cystic duct during a cholecystectomy, with resultant increased risk of bile duct injury [2,3]. This paper aims to review the current literature on Mirizzi syndrome and discuss the evolution of the diagnosis and management of this challenging condition.

### Historical Overview

This syndrome is named after the Argentinean surgeon Pablo Luis Mirizzi. Mirizzi was the first to perform an intraoperative cholangiogram in 1931 [4]. Although Mirizzi syndrome bears his name, he was not the first to describe it. It was first described by Kehr in 1905, and then, by Ruge in 1908, as partial obstruction secondary to a longstanding impacted stone and inflammation [5,6]. In 1948, Mirizzi proposed the term “hepatic duct syndrome” in the context of cholecystitis and cholelithiasis, postulating that the mechanical obstruction of the gallbladder and inflammation of the infundibulum resulted in the contraction of a “muscular sphincter” in the CHD [7]. It is now known that there is no sphincter in the hepatic duct, and that an impacted calculus causes external compression of the adjacent bile duct [4,8,9]. 

## 2. Pathophysiology of Mirizzi Syndrome

### Biliary Anatomy, Aetiology, and Risk Factors

The gallbladder consists of the fundus, body, infundibulum, and neck. The neck usually forms a gentle curve, the convexity of which forms the infundibulum. A gallstone within the infundibulum lies in close proximity to the CHD, and if sufficiently large, can cause extrinsic CHD compression with biliary obstruction. Calot’s triangle is bounded by the cystic duct inferiorly, the CHD medially, and the inferior surface of the liver superiorly [10]. Careful dissection within this anatomical space allows for the ligation and division of the cystic duct and cystic artery during cholecystectomy. However, anatomical variations in Calot’s triangle may be encountered, and dissection is made even more difficult in the presence of inflammation and adhesions, which can be severe in Mirizzi syndrome. The “critical view of safety” strategy proposed by Strasberg et al. [11] has been widely adopted to minimise the incidence of bile duct injury during laparoscopic cholecystectomy. This is based on the principle of clearing the Calot’s triangle of fat and fibrous tissue, allowing for visualisation of the cystic duct and cystic artery as two separate structures prior to their transection.

The aetiology of Mirizzi syndrome is believed to be due to chronic gallbladder inflammation and extrinsic pressure on the CHD with a background of longstanding gallstone disease. Reluctance to seek medical advice, the management of biliary colic with proton pump inhibitors according to dyspepsia of a gastroduodenal origin, or hesitation to agree with the surgical recommendation of a cholecystectomy are common factors which increase the likelihood of Mirizzi syndrome [12]. With persistence of the gallstone, pressure necrosis ensues with eventual mucosal ulceration, erosion, and fistulation in adjacent structures such as the CBD (cholecystocholedochal fistula) or CHD (cholecystohepatic fistula), or organs like the duodenum (cholecystoduodenal fistula) [1,7,13,14]. In our opinion, cholecystocolonic fistulas do not generally occur in the presence of an impacted stone. Rather, they occur in the setting of perforated cholecystitis with chronic inflammation in the body or fundus of the gallbladder while the hepatic flexure of the colon tries to wrap over, in an attempt to restrict the spread of sepsis. Jaundice is not usually associated with it. 

Congenital anatomic variations in the cystic duct are common (occurring in 18–23% of cases) and predispose patients to the development of Mirizzi syndrome [15,16,17]. Examples of such anatomical predispositions include a long cystic duct running parallel to the CBD with low insertion, a short cystic duct, or side-by-side location of the cystic and common hepatic ducts [5,9,15]. Post-cholecystectomy residual cystic duct stones have also been described as a rare aetiology [18,19]. With the increasing adoption of bail-out strategies, the incidence of retained stones in the remnant neo-gallbladder can also present as Mirizzi syndrome. Thus, Mirizzi syndrome encompasses a wide variety of clinical and pathological entities, and hence, it is essential to classify it to guide appropriate clinical management.

## 3. Classification Systems for Mirizzi Syndrome

### 3.1. McSherry Classification

In 1982, McSherry et al. [20] classified Mirizzi syndrome based on data from endoscopic retrograde cholangiopancreatography and found that constant pressure from within could result in a fistula between the gallbladder and the bile duct. The syndrome was divided into two groups. Type I is characterised by simple external compression on the CHD by impacted gallstones in the cystic duct or Hartmann’s pouch. Type II involves a similar mechanism to type I, but also includes erosion of the calculus into the CHD from the cystic duct, forming a cholecystobiliary fistula. In essence, Type II simply represents an extension of type I patients who remain untreated for any reason and in whom the disease pathology progresses with time. 

### 3.2. Csendes Classification

In 1989, Csendes et al. [13] further expanded the classification of Mirizzi syndrome into four categories by refining McSherry’s classification of type II into three further types. Csendes type I is similar to McSherry type I and refers to external compression of the bile duct by an impacted gallstone in the infundibulum or cystic duct (the original Mirizzi syndrome). However, Csendes type II refers to the presence of a cholecystobiliary fistula due to the erosion of impacted gallstones into the CBD, involving less than one-third of the circumference of the CBD. Type III refers to a cholecystobiliary fistula involving up to two-thirds of the bile duct circumference. Lastly, type IV refers to a cholecystobiliary fistula with complete destruction of the entire wall of the CBD. 

In 2007, Csendes added a further type (type V) to the classification system, which was later validated by Beltran and Csendes in 2008 [7]. Previously, they had reported a patient with both Mirizzi syndrome and gallstone ileus, which led to the conclusion that the natural history of Mirizzi syndrome may not end with just a cholecystobiliary fistula. The continuous inflammation in the area of Calot’s triangle may result in a complex fistula involving not just the biliary tract but also the adjacent viscera, via similar pathological processes [14]. Therefore, Csendes type V is defined by the presence of a cholecystoenteric fistula. Type V is further divided into type Va (without gallstone ileus) and type Vb (with gallstone ileus). The Csendes classification is summarised in Table 1.

To date, the Csendes classification remains the most widely adopted, and will be referred to in the rest of this paper; However, the authors are of the view that it is not possible with routine preoperative imaging to distinguish how much of the wall of the bile duct is destroyed with fistulation, and thus, Type II, III, and IV are more likely diagnosed intraoperatively rather than preoperatively, and hence, have minimal bearing on the informed consent process for shared decision making in treatment planning. Of the types of Mirizzi syndrome described by Csendes, type I and II are the most common [1,21,22]. Other types of Mirizzi syndrome are relatively low in incidence. An exception is type V, which may be present in up to 29% of patients with any other type of Mirizzi syndrome [1].

### 3.3. Other Classification Systems

Various other classification systems for Mirizzi syndrome exist. In 1975, prior to the recognition of external compression of the bile duct and cholecystobiliary fistula as different stages of the same disease process, Corlette et al. [23] classified cholecystocholedochal fistulas into two types—type I was described as when the fistula involved both Hartmann’s pouch and the bile duct, while type II was classified as the erosion of the cystic duct into the common bile duct by the gallstone. Starling and Matallana [24] divided type I Mirizzi syndrome into two subtypes—type Ia (long cystic duct) and type Ib (short cystic duct). 

In 2012, Beltran [9] proposed a simplified classification which categorized Mirizzi syndrome into three types. Mirizzi Ⅰ involves external compression of the bile duct by a chronic or acute inflammatory process and a gallstone impacted at the Hartmann pouch or infundibulum (corresponds to type I Mirizzi syndrome based on both classification systems by McSherry and Csendes). Mirizzi Ⅱa refers to a cholecystobiliary fistula less than 50% of the diameter of the bile duct, while Mirizzi Ⅱb refers to a cholecystobiliary fistula more than 50% of the diameter of the bile duct. Mirizzi Ⅲa refers to a cholecystobiliary fistula associated with a concurrent cholecystoenteric fistula without gallstone ileus, while Mirizzi Ⅲb has gallstone ileus. 

Paya-Llorente et al. [25] proposed a classification system modified from Beltran’s, where type I corresponds to external compression of the bile duct, type II corresponds to a cholecystobiliary fistula less than 50% of the diameter of the bile duct, and type III refers to the cholecystobiliary fistula being more than 50% of the diameter of the bile duct. Each type is further categorised into three subtypes—subtype A in which no cholecystoenteric fistula is observed, subtype B in which a cholecystoenteric fistula is present without gallstone ileus, and subtype C in which the cholecystoenteric fistula is associated with gallstone ileus. The authors believe that such a classification system will help guide the management of both the biliary and enteric aspects of Mirizzi syndrome. In conclusion, this classification systems take into account the obstruction of the CBD or CHD, the fistulation of the CBD, CHD, or duodenum, and gallstone ileus. In our opinion, patients with gallstone ileus present with intestinal obstruction and deranged pathophysiology, and have a large, well-formed, wide-mouthed fistula track between the gallbladder and the duodenum. Hence, the priority in management is not to perform cholecystectomy or manage the fistula, but instead, to manage the intestinal obstruction to restore the physiology. 

## 4. Clinical Presentation

The mean age at presentation of Mirizzi syndrome ranges from 53 to 70 years, with a female preponderance of 70% of cases [1,2,9,13,26,27,28]. Patients typically have had a longstanding history of gallstones prior to onset [1], and the syndrome may present either acutely or more commonly in a chronic manner [9,29]. The most common presentation is obstructive jaundice [5,7,8,15,27,28,29,30,31,32]. The clinical presentation ranges from asymptomatic to symptoms including right upper quadrant pain or epigastric pain, fever, nausea, vomiting, dark urine, and anorexia [9]. Patients may also present in the setting of acute cholecystitis, acute cholangitis, or pancreatitis [15,26,27,29]. A positive Murphy’s sign is present in approximately 50% of patients [33]. Mirizzi syndrome may rarely present with gallstone ileus [1,3,14]. Other less common presentations are gallbladder perforation [34] and weight loss [23]. Hepatomegaly may be present [23,24], and a hard gallbladder mass mimicking carcinoma may be palpable in 22% of patients [35]. There are no symptoms or signs that are pathognomonic for Mirizzi syndrome. 

### 4.1. Laboratory Investigations

Laboratory tests may show hyperbilirubinaemia and elevated transaminases [7,9,15,36]. Leukocytosis is frequently seen in the setting of concurrent acute cholecystitis, cholangitis, or pancreatitis [15,27,29,30]. Some studies have reported an elevated carbohydrate antigen 19-9 (CA 19-9) level, especially in patients with Mirizzi syndrome type II or higher [37,38,39]. CA 19-9 is a glycoprotein expressed by several cancers, including those originating from the pancreas, biliary tract, or stomach. CA 19-9 is also expressed by the normal pancreas, bile ductal epithelial cells, the salivary mucosa, and the meconium [40]. Hence, elevated serum CA 19-9 levels must be interpreted with caution in cases of biliary obstruction, to avoid incorrectly labelling patients as having biliary tract or gallbladder cancer when their condition is benign. In our opinion, there is no clinical value in measuring CA19-9 routinely in patients with biliary sepsis.

### 4.2. Differential Diagnosis

The presenting symptoms of right upper quadrant pain and fever may be similar to those of cholecystitis. If jaundice is present, Mirizzi syndrome may also be mistaken for other causes of obstructive jaundice, such as choledocholithiasis and cholangitis [7,9]. A malignant aetiology for obstructive jaundice also needs to be excluded, such as gallbladder carcinoma, cholangiocarcinoma, and head of pancreas cancer [15]. The treatment planning for benign biliary pathologies is widely deviant from that for malignant conditions, and thus, the accuracy of diagnosis is the Achille’s heel of appropriate clinical management. 

## 5. Diagnosis of Mirizzi Syndrome

**Figure 1:**
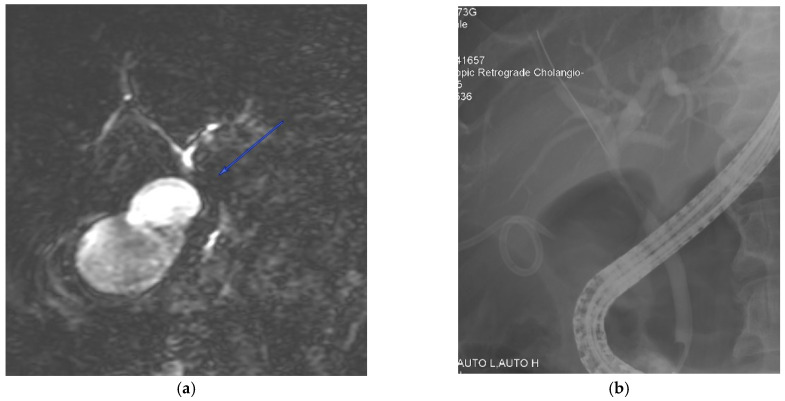
A 72-year-old male was admitted with a clinical presentation suggestive of acute cholangitis. (**a**) Magnetic resonance imaging demonstrates a hilar stricture (labelled) with suspicion of cholangiocarcinoma. In view of concomitant cholecystitis and the need to evaluate for suspected malignancy, percutaneous gallbladder drainage was performed. (**b**) A percutaneous cholecystostomy tube is visible, and an endoscopic cholangiogram shows a smooth biliary stricture with brushings that do not show malignant cells. The patient was managed with open cholecystectomy, choledochectomy, and hepaticojejunostomy. The final histology was a benign biliary structure with cholecystitis.

Obtaining a diagnosis of Mirizzi syndrome is crucial in a preoperative setting as it has a major impact on management, morbidity, and mortality [7,8,33,41]. The incidence of bile duct injuries in patients operated on with undiagnosed Mirizzi syndrome can be as high as 17% [8]. However, obtaining a preoperative diagnosis can be difficult and it can be made in only 8% to 62.5% of patients [30,31,42]. This statistic was shown to increase to 85.9% [21] when both magnetic resonance cholangiopancreatography (MRCP) and endoscopic retrograde cholangiopancreatography (ERCP) were used in combination. A combination of both MRCP and computed tomography (CT) yielded a sensitivity of 96%, specificity of 93.5%, and diagnostic accuracy of 94% [43]. Hence, most surgeons prefer to use at least two diagnostic modalities in combination [5,21]. However, this practice has not been validated by evidence [44]. Figure 1 presents a case vignette with the aid of MRCP and ERCP images, illustrating the difficulty in differentiating Mirizzi syndrome from other pathologies such as biliary malignancy.

### 5.1. Ultrasonography

Findings on an ultrasound examination of the hepato-biliary system may include an atrophic gallbladder with thick or extremely thick walls, with one large or multiple small impacted gallstones in the infundibulum or cystic duct [45]. Other findings include a dilated intrahepatic biliary tree and CHD above the level of obstruction, with a CBD of normal diameter [8,15,21,27,45]. The sensitivity of ultrasound examination ranges from 8 to 57% [7,8,9,22,46,47], with a reported diagnostic accuracy of 29% [30]. Regional inflammation and bowel gas may further limit the sensitivity of the study. Despite the low diagnostic accuracy of ultrasonography, it is often used as a cost-effective preliminary routine investigation, as Mirizzi syndrome is not always suspected even in patients with jaundice [5,21]. 

### 5.2. Computed Tomography (CT)

CT scans are useful to rule out hepatic or biliary malignancies in the porta hepatis, or other aetiologies of biliary obstruction [8,9,21,36]. It is a suitable modality for evaluating extraluminal causes of biliary compression. However, there are no specific CT findings for Mirizzi syndrome. CT is reported to have a 31–50% sensitivity [7,22,46,48]. A limitation of CT is that it often does not demonstrate biliary calculi [49]. The presence of periductal inflammation can be misinterpreted as carcinoma of the gallbladder [15]. In our opinion, though CT scans are not optimal to detect stones, they adequately visualise inflammatory fat stranding, ductal dilatation, and gallbladder wall thickening. In addition, the scan provides an idea of the dimensions of the gallbladder with some perspective on the chronicity, fibrosis, and shrunken-ness of the gallbladder, which enables us to predict technical difficulty. Aerobilia, if evident, without previous biliary instrumentation suggests cholecystobiliary or cholecystoenteric fistulation. 

### 5.3. Magnetic Resonance Cholangiopancreatography (MRCP)

**Figure 2:**
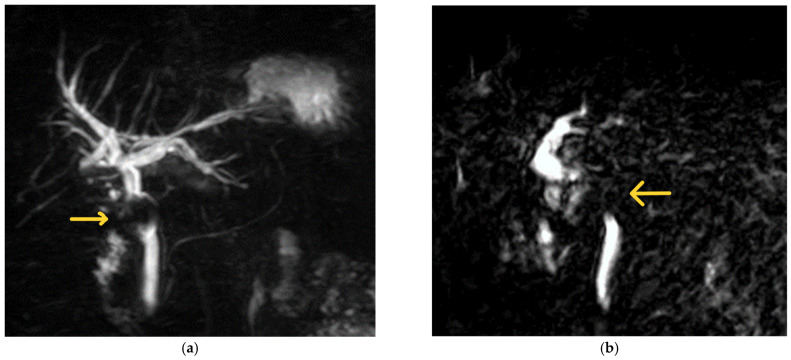
Examples of MRCP findings in Mirizzi syndrome. (**a**) The first MRCP shows a filling defect (labelled) caudal to the confluence of the hepatic ducts with upstream biliary dilatation. The patient was a 67-year-old female with a background of Mirizzi syndrome treated with laparoscopic subtotal cholecystectomy at another hospital 10 months prior. She was subsequently diagnosed with a retained 19 mm cystic duct stone with a fistula in the CHD (Csendes Type III Mirizzi syndrome) and underwent laparoscopic CBD exploration with primary repair at our institution. (**b**) The second image illustrates an abrupt cut-off at the level of the CHD with a filling defect (labelled). This was taken from a 70-year-old man whose initial presentation of Mirizzi syndrome was that of acute pyogenic cholangitis. He subsequently underwent ERCP with biliary stenting, followed by laparoscopic CBD exploration, biliary stent removal, and primary closure of the CBD 8 weeks later.

MRCP is the preferred imaging modality to assess biliary anatomy and the presence of biliary calculi. Although the reported diagnostic accuracy of MRCP is 50% [30], it has a sensitivity of 77.8–100% in the diagnosis of Mirizzi syndrome [8,16,27,46,50]. It is regarded as the preferred non-invasive imaging modality for the diagnosis of Mirizzi syndrome [22]. MRCP findings in Mirizzi syndrome include dilatation of the intrahepatic bile ducts, narrowing of the CHD, and gallstone(s) in the cystic duct [49]. MRCP can also rule out other causes of bile duct obstruction, such as choledocholithiasis, or be used to assess inflammation around the gallbladder [9]. Though MRCP is a non-invasive modality, ERCP has increased accuracy to diagnose a cholecystobiliary fistula [51]. MRCP images from patients with Mirizzi syndrome are shown in Figure 2 for illustration.

### 5.4. Endoscopic Retrograde Cholangiopancreatography (ERCP)

**Figure 3:**
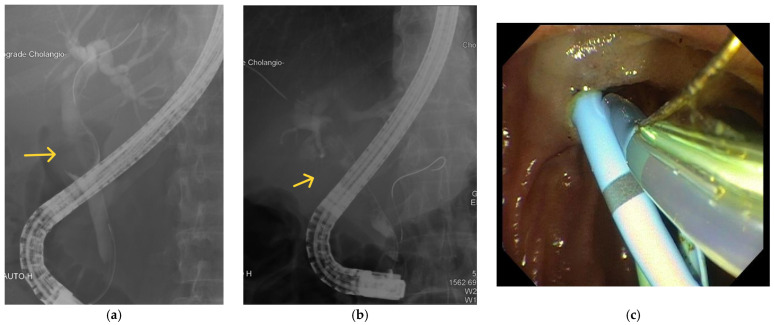
Examples of ERCP findings in Mirizzi syndrome. (**a**) There is a filling defect (labelled) seen in the CBD. (**b**) There is a filling defect (labelled) in the CHD. (**c**) Biliary and pancreatic stenting during ERCP.

Despite its invasive nature, ERCP is still the gold standard for the diagnosis of Mirizzi syndrome. ERCP has a sensitivity of 50–100% [3,30,46,50], with a diagnostic accuracy of 55–90% [30,42]. ERCP provides superior visualisation of the extrahepatic bile ducts, and can identify extrinsic biliary compression and the presence of a cholecystobiliary or cholecystoenteric fistula [5,36]. Figure 3 illustrates examples of images taken using ERCP. Biliary stenting or stone retrieval can also be offered to relieve obstruction, giving ERCP the advantage of being both a diagnostic and therapeutic procedure [51]. An example of biliary and pancreatic stenting during ERCP is shown in Figure 3c. The use of endoscopic ultrasound prior to ERCP to further evaluate the bile ducts and pancreas was demonstrated in a patient with Mirizzi type I by Rayapudi et al. [52], while some studies have used intraductal ultrasonography during ERCP to delineate defects in the ductal mucosa to localise cholecystobiliary fistulas and to differentiate causes of biliary strictures [53,54].

The main disadvantage of ERCP is the risk of major complications. These include pancreatitis (3.5%), haemorrhage (1.3%), cholangitis (1%), perforation (0.1–0.6%), and the associated cardiopulmonary complications of sedation [55]. In our opinion, ERCP is best reserved as a therapeutic modality to clear biliary stone disease burden, decompress the biliary system in the presence of sepsis, and obtain biliary brushings or cytology in patients who have strictures which are suspected to be malignant.

### 5.5. Percutaneous Transhepatic Cholangiography (PTC)

PTC may be performed as a diagnostic investigation, usually in cases where ERCP failed [5]. Percutaneous biliary drainage can be performed in the same setting. PTC can also be used in cases with previous bilioenteric anastomosis or intrahepatic dilation with a high obstruction [56]. However, PTC is rarely used as a diagnostic tool. 

## 6. Management of Mirizzi Syndrome

### 6.1. Surgery 

Surgery is the mainstay of therapy for Mirizzi syndrome, because the gallbladder must be removed not only because it contains the stone, but also because it forms them! However, surgery for Mirizzi syndrome is technically difficult and presents a challenge to any surgeon’s ability and dexterity [14]. Many patients are only diagnosed with Mirizzi syndrome intraoperatively [30]. The intraoperative findings may include an oedematous or atrophic gallbladder, an impacted gallstone or multiple gallstones in the infundibulum or the gallbladder neck, dense fibrosis with obliteration of Calot’s triangle, and dense adhesions in the subhepatic space [5]. The severe inflammatory process with dense adhesions and associated oedematous tissues distorts the anatomy, increasing the risk of bile duct injury [31,57,58,59,60]. 

#### 6.1.1. Cholecystectomy 

The various types of Mirizzi syndrome warrant different surgical approaches, depending on the classification, intraoperative findings, and level of expertise [60]. Traditionally, open cholecystectomy was recommended for Mirizzi syndrome [36]. Open surgery allows the use of proprioception or touch of the surgeon’s hand, even though difficult surgery is not necessarily easier or safer when performed in an open manner [61]. Total cholecystectomy is feasible in Mirizzi syndrome type I, and for some cases of type II and III, depending on the severity of inflammation and anatomical distortion within Calot’s triangle [5,6,50,62]. A fundus-first approach to cholecystectomy is often recommended, entailing a dissection from the fundus of the gallbladder toward Hartmann’s pouch [8,32,50,63]. If significant adhesions are encountered in Calot’s triangle, a bail-out option of subtotal cholecystectomy can be considered, and this approach has been shown to be safe and feasible [6,15,64]. Subtotal cholecystectomy may either be of the fenestrating or reconstituting subtype, depending on whether the lower end of the gallbladder is left open with suture closure of the cystic duct internally or closed to create a remnant gallbladder, respectively [65]. Although reconstituting subtotal cholecystectomy is shown to have better outcomes overall, the fenestrating technique still has its utility if the closure of the gallbladder stump is not possible, and both methods should be seen as complementary [66]. If leakage of bile from the CBD is observed following extraction of an impacted stone in the cystic duct, a cholecystobiliary fistula is strongly suspected [9,32,50]. In our opinion, Hartmann’s pouch can be safely closed provided the common hepatic duct is not narrowed, or else bilioenteric anastomosis is warranted. 

Both laparoscopic cholecystectomy and laparoscopic subtotal cholecystectomy have been described for Mirizzi syndrome. In 1992, Paul et al. [67] reported the first successful laparoscopic surgery as a treatment for Mirizzi syndrome type I, with the use of choledochoscopy. The laparoscopic approach can be technically challenging due to inflammation and the obliteration of Calot’s triangle, making dissection of the cystic duct and cystic artery hazardous [2,3,56]. It has been observed in some studies that the chances of bile duct injury and conversion can be as high as 14% and 30%, respectively [68,69]. Cui et al. [21] reported a 64.6% rate of conversion to open surgery among 65 patients with Mirizzi syndrome type I. In 2009, a systematic review by Antoniou et al. [3] showed an overall complication rate of 16%, conversion rate of 41%, and mortality rate of 0.8% for laparoscopic cholecystectomy in the setting of Mirizzi syndrome; consequently, the authors did not recommend the laparoscopic approach. It is our practice to offer the laparoscopic approach routinely as “conversion is not a crime [70]”, and with the adoption of subtotal cholecystectomy, the success of the laparoscopic approach is high. Despite the technical difficulties of the laparoscopic approach, it can still be safe and feasible in experienced hands, especially for Mirizzi syndrome type I [46,71,72,73]. Laparoscopic subtotal cholecystectomy is a feasible approach to reducing the risk of bile duct injury, in particular for cases with a cholecystobiliary fistula [41]. Ibrarullah et al. [34] suggested that for Mirizzi type I, a trial laparoscopic dissection is reasonable for an experienced surgeon, with a low threshold to convert to an open procedure. A more recent case series by Shirah et al. [33] reported no postoperative complications in 49 patients with Mirizzi syndrome who underwent laparoscopic cholecystectomy, with an open conversion risk of 8%. 

Notably, Rohatgi and Singh [56] described a step-by-step approach to the laparoscopic management of Mirizzi syndrome types I and II, which emphasises opening the gallbladder at the fundus and removal of stones within the fistula from that approach, in order to facilitate demonstration of the neck of the gallbladder and allow subtotal cholecystectomy to be performed safely. They also emphasise the importance of intraoperative cholangiography. Their approach is similar to the “inside approach of the gallbladder” described by Hubert et al. in the context of subtotal cholecystectomy [74]. Some experts have also suggested that laparoscopic management is safe in Mirizzi syndrome types II and III, especially if there has been preoperative endoscopic biliary stenting [68,75,76,77]. A systematic review by Zhao et al. [75] in 2020 showed that for patients with Mirizzi syndrome treated laparoscopically, the overall complication rate was 6% and the conversion rate 34%, with no mortality. The outcomes were significantly better in the presence of a preoperative diagnosis. The results of their study, which included patients with Mirizzi syndrome types I to IV, compared with those of Antoniou et al. [3], show a trend of improving outcomes of laparoscopic approaches in Mirizzi syndrome, especially for patients with types I and II, who formed the majority in their study. Moreover, a study by Nag and Nekarakanti [78] comparing laparoscopic and open surgical approaches in patients with Mirizzi syndrome (types I to III) showed that laparoscopic management is noninferior, with reduced surgical blood loss and lengths of hospital stay. 

It is recommended to use frozen section of the gallbladder wall to rule out coexistent gallbladder carcinoma [79]. If a diagnosis of surgically resectable gallbladder carcinoma is made, a radical cholecystectomy with hepatic wedge resection and lymph node dissection must be performed [9]. Prasad et al. [80] reported a five times higher incidence of gallbladder carcinoma in patients with Mirizzi syndrome as compared to patients with uncomplicated gallstone disease. They found no distinguishing features between Mirizzi syndrome and gallbladder carcinoma clinically, and the diagnosis of gallbladder carcinoma was made on final histology. Since Mirizzi syndrome and gallbladder carcinoma share the same risk factor of longstanding gallstone disease with stasis, Mirizzi syndrome itself should be considered a risk factor for gallbladder carcinoma [80,81]. In our opinion, the decision for radical cholecystectomy must be communicated to the patient pre-operatively, and this shared decision making is only possible if high-quality imaging is obtained and a diagnosis is suspected. In the absence of a clear preoperative diagnosis and informed consent, a completion cholecystectomy should be performed on a separate occasion or else a surgeon risks a professional negligence charge [82]. 

In general, cholecystectomy is considered a “general surgical” operation and widely performed by general and acute care surgeons despite the global recognition of hepato-biliary surgery as a distinct specialty. It is not the purpose of our review to recommend or suggest that all gallbladders should be referred to hepato-biliary surgeons, but we advocate that once a diagnosis of Mirizzi syndrome is established, a referral and transfer of the patient to a trained hepato-biliary surgeon should be considered if not outright warranted or recommended. This view is not intended to undermine the technical abilities of a trained general surgeon, but it considers the importance of decision making to determine the procedure of choice in a given situation and the management of any possible postoperative complications. 

#### 6.1.2. Intraoperative Imaging

Intraoperative cholangiography (IOC) can be useful to determine the location and size of a cholecystobiliary fistula, detect bile duct stones, and verify the integrity of the bile duct wall, as well as retrieve residual stones in a postoperative setting. However, IOC may be difficult to perform, and there is risk of bile duct injury due to the distorted anatomy commonly encountered in Calot’s triangle [8,9].

On the other hand, intraoperative ultrasonography has proven useful in identifying the anatomy of the biliary tree, and aids in the precise dissection of the bile duct in the presence of inflammation [8,27,56]. Laparoscopic ultrasonography provides real-time multiplanar images of the bile ducts from multiple angles. It is recommended to be used especially when there has been no preoperative diagnosis of Mirizzi syndrome or when the syndrome is suspected intraoperatively [7].

Near-infrared fluorescence cholangiography using indocyanine green (ICG) dye is a new technique for visualising the bile duct during laparoscopic cholecystectomy. In 2010, Ishizawa et al. [83] first described the use of fluorescent cholangiography to identify biliary anatomy in real time during the dissection of Calot’s triangle. Fluorescence cholangiography is a safe and effective technique which significantly increases the visualisation rates of extrahepatic biliary structures, including the CHD and CBD [84]. A shorter operating time, the absence of a need to cannulate the cystic duct, and the absence of radiation exposure are some of the advantages of ICG fluorescence cholangiography as compared to IOC [85]. Nitta et al. [86] reported its use in a patient with acute cholecystitis and Mirizzi syndrome type I. There is a lack of literature on the use of ICG fluorescence cholangiography in Mirizzi syndrome. In our view, ICG dye is good for visualizing the extrahepatic biliary tree but not the intrahepatic biliary tree, and thus, the exact role of ICG application needs to be better defined by more evidence. Considering that Mirizzi syndrome is uncommon, and ICG capabilities are not widely available, the evidence is expected to be slow and low-quality due to difficulty in conducting prospective multicentre randomised studies. 

In our opinion, intraoperative imaging should be performed with the intent to facilitate surgery, and preoperative imaging should be the mainstay for establishing diagnoses, determining severity, management planning, and guiding surgical decisions. However, when a preoperative diagnosis is not established due to vague clinical presentation or a lack of good quality in the imaging test, intraoperative imaging is useful to guide decision making. 

#### 6.1.3. Choledochoplasty

If a subtotal cholecystectomy has been performed, a cuff of the gallbladder or cystic duct is left behind, which can be used to repair the cholecystocholedochal fistula, a technique used in choledochoplasty. Repair of the fistula can be achieved through primary closure or through choledochoplasty with the gallbladder cuff, with or without T-tube placement in the CBD [6,50,56]. This may be performed for Mirizzi syndrome type II and selected cases of type III [21,44,50,63,87]. In terms of the size of the cuff of the gallbladder for choledochoplasty, some authors recommend a 5 mm cuff for patients with fistula sizes of less than one-third of the CBD diameter (type II), and a 10 mm cuff for patients with fistula sizes between one-third and two-thirds of the diameter of the CBD (type III) [59,88]. However, Chen et al. [5] suggested that bilioenteric anastomosis is a preferred alternative. This is especially so in the presence of bile duct ischaemia or if the defect is large [63]. Kumar et al. [47] reported no significant difference in overall morbidity between choledochoplasty and bilioenteric anastomosis. In our experience, for laparoscopic subtotal cholecystectomy using barbed sutures to create a neo-gallbladder and bile duct exploration with primary repair, both techniques are safe and feasible, and 3D laparoscopy adds to surgical precision with better depth perception that facilitates intracorporeal suturing [89]. Also, for primary repair of the bile duct for choledochoplasty, the size of the gallbladder cuff should be adequate to ensure that there is no tension on the closure and ensure it is not too redundant. If the bile duct is dilated and can be closed primarily, we do not advocate choledochoplasty, and the vascularity of the gallbladder cuff cannot be guaranteed. 

#### 6.1.4. T-Tube (Kehr Tube)

If a cholecystobiliary fistula (Mirizzi syndrome type II or higher) is encountered, CBD exploration is routinely undertaken (either through open surgery or laparoscopically), as CBD stones are found in 25–40% of cases of Mirizzi syndrome [9]. The bile duct is explored through a different incision over the CHD or CBD, with placement of a T-tube drain to protect the CBD suture [13,24,27,31,42,88]. Csendes et al. [13] cautioned against placing the T-tube through the fistula, as bile leak can occur readily and a benign stricture can appear late after surgery. In cases when the quality of tissue repair is in doubt, T-tube insertion not only serves to decompress the bile duct, but also shapes the duct in order to minimise the risk of bile leakage. Furthermore, a T-tube offers a route of access for cholangiography before concluding the surgery, and also for the future removal of retained stones [5,27,30,58,59]. In our experience, the use of T-tubes is on the decline, with only anecdotal use at our institution. This is largely due to the local policy on laparoscopic subtotal cholecystectomy or laparoscopic bile duct exploration with primary repair without a T-tube or abdominal drains. Intraoperative choledochoscopy provides good views, and biliary clearance can be achieved from second-order biliary radicles cranially to the duodenum caudally under direct visualization, thus increasing confidence for primary closure. Along with increased familiarity with intracorporeal suturing and the refinement of skills, the routine use of T-tubes is largely unnecessary, and it may serve to increase the procedure’s association with morbidity rather than its clinical value. However, in resource-limited situations or when the skillset for laparoscopic biliary surgery is not available, the T-tube remains a valuable adjunct. 

#### 6.1.5. Bilioenteric Anastomosis (Hepaticojejunostomy)

In patients with a cholecystobiliary fistula with a background of chronic inflammation, the risk of stricture development increases; therefore, bilioenteric anastomosis is recommended [13,21,30,31,57,58,60,90]. Options include choledochoduodenostomy or Roux-en-Y hepaticojejunostomy, with hepaticojejunostomy being the safer option [63]. Choledochoduodenostomy should be avoided in the absence of an adequately dilated CBD [33]. The consensus is that cholecystectomy with bile duct excision and reconstruction with hepaticojejunostomy is recommended for Mirizzi syndrome type IV [5,13,30,31,50,58,59,60,63,88]. Some authors also recommend cholecystectomy with hepaticojejunostomy as the preferred option for type III [47,50,63,91]. In our opinion, if the bile duct is dilated enough for a safe primary repair without the risk of narrowing, primary repair should be performed to reduce the morbidity associated with hepaticojejunostomy. 

For the treatment of Mirizzi syndrome type Va, laparotomy is the safest method [5], and involves division of the cholecystoenteric fistula, cholecystectomy (either total or subtotal), and closure of the fistula [36]. The treatment of type Ⅴb is more controversial. It is advisable to treat the gallstone ileus first, and subsequently, after a certain duration of recovery (three or more months), reassess the need for cholecystectomy or treatment of the cholecystobiliary fistula [14]. In our experience, once a fistula is developed, and intestinal obstruction from gallstone ileus is surgically managed, the gallbladder can be safely left behind as a gallbladder without risk of biliary events. This strategy reduces the surgical risk in classical gallstone ileus patients who are elderly with multiple comorbidities, and “doing less is more” has served us well. The fistula serves as an alternative path for gallbladder drainage into the duodenum with the natural antegrade expulsion of remnant gallstones. 

### 6.2. The Role of Endoscopic Interventions

ERCP can be used as an adjunct to surgical treatment, though definitive treatment still necessitates removal of the gallbladder through surgery. ERCP allows for a sphincterotomy for stone extraction and facilitates other interventions such as the placement of stents or a nasobiliary tube [29,30,42,58,60]. Patients who may stand to benefit from ERCP include poor surgical candidates, or those with acute cholangitis in whom biliary decompression is a temporizing measure before definitive surgery [9]. 

Furthermore, ERCP with the placement of one or more biliary stents can facilitate cholecystectomy or the closure of a cholecystobiliary fistula, by ensuring the patency of the CBD [92]. A combined approach with laparoscopic surgery and ERCP offers potential for a minimally invasive method of treating Mirizzi syndrome. Li et al. [93] treated 27 patients with Mirizzi syndrome (16 type I, 5 type II, and 6 type III) with a “tripartite” combination of ERCP, laparoscopy, and choledochoscopy. When compared to patients who had undergone routine laparotomy, they reported a similar rate of postoperative complications (7%), but a shorter operation time, lower intraoperative blood loss, earlier initiation of enteral nutrition, and a decreased length of hospital stay. In their study, the endoscopic nasobiliary drainage tube placed during ERCP also enabled the use of nasobiliary drainage cholangiography postoperatively. 

**Figure 4:**
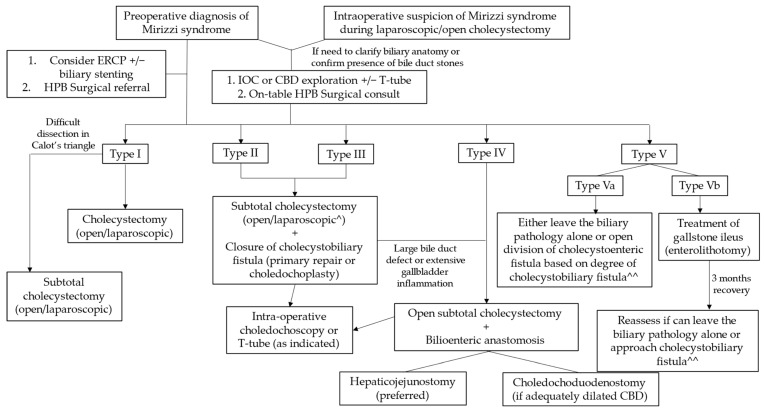
Management of Mirizzi syndrome according to Csendes classification. ^ Laparoscopic approach reserved for institutions with the necessary surgical expertise, with a low threshold for conversion to open surgery. ^^ Treatment according to the presence or absence and degree of cholecystobiliary fistula is depicted under types I–IV.

A proposed management algorithm for Mirizzi syndrome is shown in Figure 4. 

## 7. Future Perspectives and Emerging Treatments

### 7.1. Advances in Minimally Invasive Surgery

As laparoscopic techniques continue to evolve, along with technological advances, the laparoscopic approach remains a possibility for more severe types of Mirizzi syndrome, given the right expertise and careful patient selection. Yetisir et al. [94] described a patient with Mirizzi syndrome type Va in whom laparoscopic subtotal cholecystectomy and resection of the cholecystocolic fistula were performed with the help of Tri-Staple. The postoperative course was uneventful, and the patient remained symptom-free at the eight-month follow-up. Recently, Gomez et al. [95] successfully performed laparoscopic cholecystectomy and fistula resection with primary closure in 16 patients with Mirizzi syndrome type Va. 

Laparoscopic transfistulous bile duct exploration has been proposed as a safe approach for patients with Mirizzi syndrome. In 2016, Chuang et al. [96] introduced the concept, using a fundus-first dissection and direct infundibulotomy to approach the impacted stone and fistula, and also a subtotal cholecystectomy. This technique was performed for patients with type II or IV Mirizzi syndrome. The authors also successfully performed single-incision laparoscopic transfistulous bile duct exploration and subtotal cholecystectomy for three patients with Mirizzi syndrome type II. Recently, Nassar et al. [97] demonstrated the use of laparoscopic transfistulous bile duct exploration in the management of patients with Mirizzi syndrome type II, by using the cholecystocholedochal fistula to access the bile duct via choledochoscopy. Senra et al. [76] used a similar approach via infundibulotomy to access the CBD with choledochoscopy for laparoscopic CBD exploration in patients with type II and above Mirizzi syndrome, in order to avoid fistulotomy or choledochotomy. Hence, it was not necessary to obtain a critical view of safety, avoiding potentially hazardous dissection in Calot’s triangle. For large stones, the authors augmented their approach with the laser-assisted bile duct exploration using the laparoendoscopy (LABEL) technique. The LABEL technique allows large impacted CBD stones to be fragmented into smaller pieces, facilitating stone extraction during laparoscopic CBD exploration and minimising the need for choledochotomy [98]. In our opinion, a longitudinal incision on the bile duct to achieve stone expulsion by manipulating the laparoscopic graspers is safe and sufficient, and laser fragmentation is mostly unnecessary. Bail-out cholecystectomy strategies such as subtotal cholecystectomy, the liberal adoption of good-quality imaging for better anatomical details to guide surgical care, access to intra-operative choledochoscopy [99], and technical advances in absorbable barbed sutures, which have facilitated intracorporeal suturing [89], have facilitated the minimal access management of Mirizzi syndrome patients.

#### 7.1.1. Single-Incision Laparoscopic Surgery (SILS)

SILS has emerged as an alternative method to standard multiport laparoscopic surgery, as part of the effort to minimise complications associated with multiple incisions and improve cosmesis. The indication for single-incision laparoscopic cholecystectomy has expanded to include more complicated gallbladder diseases, and Chang et al. [100] reported two successful cases of single-incision laparoscopic cholecystectomy for patients with Mirizzi syndrome types I and II without significant morbidity. In 2022, Chuang et al. [101] studied the outcomes between single-incision laparoscopic transfistulous bile duct exploration and four-incision laparoscopic transfistulous bile duct exploration, and found that the outcomes were similar for patients with Mirizzi syndrome types II, III, and IV. In our opinion, SILS increases the risk of incisional port site hernia, and hence, has to be carefully recommended to patients outside units with substantial expertise in this technique. SILS increases technical difficulty due to the loss of triangulation, and in an already complex situation, it increases technical difficulty, so we are of the opinion that SILS should not have a place outside the domains of exemplary experts or as a part of research protocol. 

#### 7.1.2. Robotic-Assisted Surgery

Robotic-assisted approaches to cholecystectomy have been described in the literature [102]. Robotic surgery allows for more precise and finer control than laparoscopy, with the help of EndoWrist instruments. Robot-assisted systems can also provide enhanced visualisation via a three-dimensional camera [5,103]. A novel approach combining robotic-assisted cholecystectomy (either subtotal or total) and endoscopic biliary stenting has been shown to be safe and feasible, with low conversion rates and low morbidity [104,105]. According to Lee et al. [104], patients who underwent robotic-assisted subtotal cholecystectomy had significantly shorter hospital stays than those who underwent open surgery, with the only drawback being longer operation time for the robotic-assisted approach. Tung et al. [103], in particular, reported that the freedom of movement provided by EndoWrist instruments aids in performing choledochoplasty as compared to an ordinary laparoscopic approach. Furthermore, another benefit of the increased manoeuvrability of the robot, as mentioned by Magge et al. [105], is the ability to perform CBD explorations readily in a setting of Mirizzi syndrome where fibrosis and scarring make the critical view of the safety and identification of portal structures challenging. Studies describing the use of robotic-assisted surgery in the treatment of Mirizzi syndrome have been limited to patients who have undergone preoperative endoscopic biliary stenting. However, a recent large cohort study by Kalata et al. showed that for patients with benign biliary pathology, robotic-assisted cholecystectomy has a higher rate of bile duct injuries than laparoscopic cholecystectomy (0.7% vs. 0.2%) [106]. Hence, robotic-assisted cholecystectomy is not routine and should be carefully considered on a case-by-case basis. In our opinion, robotic approaches to gallbladder and bile duct exploration can be useful for individual surgeons and units to gain technical expertise and skills, which enables units and individual surgeons to perform more complex surgeries such as pancreaticoduodenal resections. The ability to obtain fluorescence imaging, the 3D view with depth perception, and the EndoWrist movements to facilitate intracorporeal suturing remain the advantages of robotic technology, and an appropriately informed patient, including regarding cost details, can be enlisted to have a robotic procedure for Mirizzi syndrome [107]. From an ethical viewpoint, a surgeon has to ensure that the patient’s best interests are considered and refrain from using the robotic platform to serve their own personal interests of gaining experience, and thus, it is recommended that early cases be enlisted and recruited after seeking approval from local institutional research ethics committees to avoid a loss of public trust from potential for conflicts of interest. 

### 7.2. Other Novel Techniques and Therapies

#### Cholangioscopy

**Figure 5:**
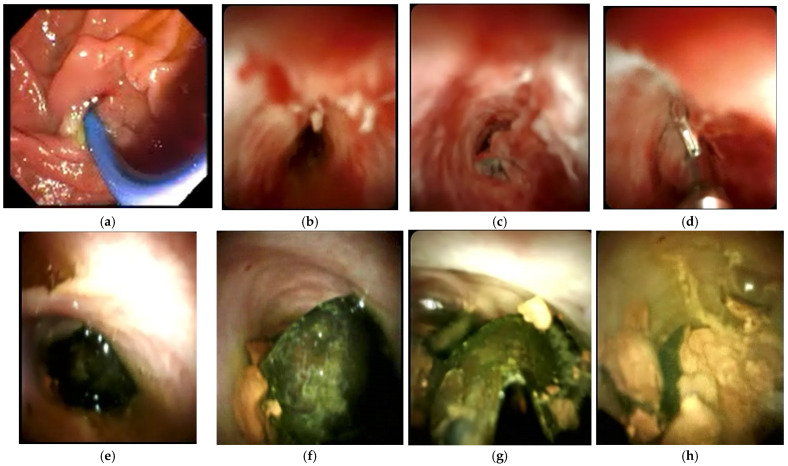
ERCP with cholangioscopy. (**a**) Endoscopic view of ampulla of Vater. (**b**) Cholangioscopy image of CBD narrowing with inflammation in a patient with Mirizzi syndrome. (**c**) CBD stricture due to cholangiocarcinoma in a patient initially suspected to have Mirizzi syndrome. (**d**) Sampling of biliary stricture using SpyBite forceps. (**e**) Black pigment of stone seen partly in the CHD and cystic duct. (**f**) Another view of the stone in (**e**). (**g**) The same stone in (**e**) being fragmented by SpyGlass electrohydraulic lithotripsy. (**h**) Fragmented stones after lithotripsy.

In a study of 34 patients, Bhandari et al. [108] reported a 100% success rate of cystic duct stone clearance with single-operator cholangioscopy-guided laser lithotripsy, with 94% of patients achieving ductal clearance in the first attempt. A larger series by Tsuyuguchi et al. [109] involving 53 patients showed that peroral cholangioscopy-directed lithotripsy was successful in stone removal in 91% of patients with Mirizzi syndrome, the majority of whom were type II. Peroral cholangioscopy has utility in distinguishing between Mirizzi syndrome type I and II preoperatively if ERCP is unable to, and also aids in stone removal for type II prior to cholecystectomy [109]. Recently, Kawai et al. [110] reported the first case of the successful complete ductal clearance of stones via single-operator cholangioscopy-guided electrohydraulic lithotripsy in a patient with Mirizzi syndrome type IV. These studies demonstrate how advances in endoscopic visualisation systems have facilitated the treatment of more difficult biliary stones. In addition, cholangioscopy aids in tissue sampling in patients where malignancy needs to be ruled out and guides care. The SpyGlass^®^ system for cholangioscopy is already in use at our institution, and Figure 5 provides illustrations of its utility in direct visualisation of biliary pathology, as well as in procedures such as biopsy and stone removal.

## 8. Conclusions

Our understanding of Mirizzi syndrome has evolved significantly since the writings of Mirizzi. Despite the many advances in diagnostic imaging modalities and surgical techniques in the treatment of complex biliary disease, the management of Mirizzi syndrome is challenging. Due to the low incidence of Mirizzi syndrome, randomised controlled trials are not performed, and retrospective studies form the bulk of scientific evidence. It must be emphasized that the treatment of Mirizzi syndrome should depend on the surgeon’s experience and available resources and capabilities. In our opinion, multidisciplinary team engagement in decision making is a key determinant of the long-term success of the management strategy. 

It is evident that laparoscopy is no longer a contraindication to the treatment of Mirizzi syndrome, even in the presence of a cholecystobiliary fistula. As with any major surgery, good patient selection is paramount. The development of advanced endoscopic techniques, both as a diagnostic modality and therapeutic tool for stone retrieval, have facilitated improved outcomes with laparoscopic surgery. 

Unfortunately for a junior surgeon, the diagnosis of Mirizzi syndrome is not straightforward, and it is often diagnosed intraoperatively. Good intraoperative decision making is crucial. If a critical view of safety cannot be obtained due to the difficult anatomy at Calot’s triangle, there should be no shame in seeking help from a more experienced surgeon, as compared to putting the patient at risk of bile duct injury. Even for experienced surgeons, setting a low threshold to resort to a bail-out procedure, such as subtotal cholecystectomy, is prudent.

## Figures and Tables

**Table 1:** Classification system for Mirizzi syndrome according to Csendes.

Type of Mirizzi Syndrome	Description (Csendes)	Schematic Representation
I	External compression of the bile duct by an impacted gallstone in the infundibulum or cystic duct	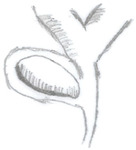
II	Cholecystobiliary fistula involving less than one-third of the circumference of the bile duct	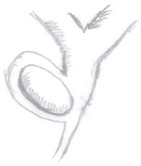
III	Cholecystobiliary fistula involving up to two-thirds of the bile duct circumference	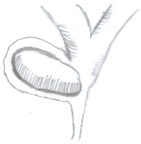
IV	Cholecystobiliary fistula with complete destruction of the entire wall of the bile duct	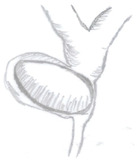
V	Presence of cholecystoenteric fistula without gallstone ileus (Va)	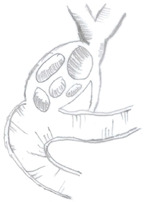
	Presence of cholecystoenteric fistula with gallstone ileus (Vb)	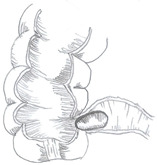

## Data Availability

Not applicable as no new data were created.
